# Effects of pelvic fixation strategies and multi-rod constructs on biomechanics of the proximal junction in long thoracolumbar posterior instrumented fusions: a finite-element analysis

**DOI:** 10.1007/s43390-024-00932-w

**Published:** 2024-08-20

**Authors:** Muzammil Mumtaz, Andrew P. Collins, Niloufar Shekouhi, Karthika Varier, Sudharshan Tripathi, Christopher P. Ames, Vedat Deviren, Aaron J. Clark, Vijay K. Goel, Alekos A. Theologis

**Affiliations:** 1 Engineering Center for Orthopedic Research Excellence (E-CORE), Departments of Bioengineering and Orthopaedic Surgery, University of Toledo, Toledo, OH USA; 2 Department of Orthopaedics and Sports Medicine, University of Washington, Seattle, WA USA; 3 Department of Neurological Surgery, UCSF, San Francisco, CA USA; 4 Department of Orthopaedic Surgery, University of California-San Francisco (UCSF), 500 Parnassus Ave, MUW 3rd Floor, San Francisco, CA 94143 USA

**Keywords:** Finite-element analysis, Biomechanics, Multi-rod constructs, Pelvic fixation, Proximal junctional kyphosis/failure

## Abstract

**Purpose:**

To assess the effect of various pelvic fixation techniques and number of rods on biomechanics of the proximal junction of long thoracolumbar posterior instrumented fusions.

**Methods:**

A validated spinopelvic finite-element (FE) model was instrumented with L5–S1 ALIF and one of the following 9 posterior instrumentation configurations: (A) one traditional iliac screw bilaterally (“2 Iliac/2 Rods”); (B) T10 to S1 (“Sacral Only”); (C) unilateral traditional iliac screw (“1 Iliac/2 Rods”); (D) one traditional iliac screw bilaterally with one midline accessory rod (“2 Iliac/3 rods”); (E) S2AI screws connected directly to the midline rods (“2 S2AI/2 Rods”); and two traditional iliac screws bilaterally with two lateral accessory rods connected to the main rods at varying locations (F1: T10–11, F2: T11–12, F3: T12–L1, F4: L1–2) (“4 Iliac/4 Rods”). Range of motions (ROM) at T10–S1 and T9–T10 were recorded and compared between models. The T9–T10 intradiscal pressures and stresses of the T9–10 disc’s annulus in addition to the von Mises stresses of the T9 and T10 vertebral bodies were recorded and compared.

**Results:**

For T10–S1 ROM, 4 iliac/4 rods had lowest ROM in flexion and extension, while 2 S2AI/2 rods showed lowest ROM in rotation. Constructs with 3 or 4 rods had lower stresses on the primary rods compared to 2-rod constructs. At the proximal adjacent disc (T9–10), 4 iliac/4 rods showed lowest ROM, lowest intradiscal pressures, and lowest annular stress in all directions (most pronounced in flexion–extension). Under flexion and extension, 4 iliac/4 rods also showed the lowest von Mises stresses on the T10 vertebral body but the highest stresses on the T9 vertebral body.

**Conclusions:**

Dual iliac screws with 4 rods across the lumbosacral junction and extending to the thoracolumbar junction demonstrated the lowest T10–S1 ROM, the lowest adjacent segment disc (T9–T10) ROM, intradiscal pressures, and annular stresses, and the lowest UIV stresses, albeit with the highest UIV + 1 stresses. Additional studies are needed to confirm whether these biomechanical findings dictate clinical outcomes and effect rates of proximal junctional kyphosis and failure.

## Introduction

When long thoracolumbar posterior instrumented constructs for adult spinal deformity (ASD) extend proximal to L3 and terminate in the sacrum, sacral fixation is biomechanically compromised [1, 2] which leads to an increased propensity for sacral fracture, sacral screw loosening and/or pull-out, and lumbosacral nonunion [3–7]. As such, for instrumented fusions extending above L3, pelvic fixation is advised [1, 2, 7–9].

Pelvic fixation can be achieved in a myriad of ways. Early instrumentation techniques, including iliosacral screws, Harrington iliac bars, sacroiliac plate, Luque–Galveston, and Chopin block, were associated with unacceptably high rates of pseudoarthrosis and screw pull-out [4, 10, 11]. Newer pelvic instrumentation techniques, including iliac screws and S2 alar-iliac (S2AI) screws, have demonstrated excellent biomechanical and clinical results. Traditionally, pelvic fixation has been achieved by placing a single iliac screw or S2AI on each side of the pelvis. More recently, there has been an increasing trend in placing two iliac bolts on one or both sides of the pelvis to facilitate creating multi-rod (≥ 3) constructs across the lumbosacral junction and lumbar spine to decrease rates of pseudoarthroses and rod breakages at the lumbosacral junction and thoracolumbar junction in long posterior thoracolumbar instrumented fusions [12–14].

While pelvic fixation has provided clear benefits with regard to protecting sacral fixation and decreasing complications at the lumbosacral junction, [15, 16], this has been mirrored by an increase in proximal junctional pathology, including proximal junctional kyphosis (PJK) and proximal junctional fractures (PJF) [17–19]. While this phenomenon is postulated to occur, in part, as a result of more rigid fixation at the base of the construct (i.e., pelvis) transmitting greater forces, stresses, and range of motion (ROM) to the proximal junction, this theory has yet to be evaluated biomechanically.

As such, the aim of this study is to assess the effect of various spinopelvic fixation techniques and multi-rod constructs on the biomechanics of the proximal junction of long thoracolumbar posterior instrumentation constructs commonly utilized to manage ASD.

## Materials and methods

A previously validated thoracolumbar model [T8–pelvis; pelvic incidence (PI) 44.5º; L4–S1 lordosis 38.5º; L1–S1 lordosis 55.7º] was used in this study [20, 21]. The validated model was developed using CT scans that were used to construct three-dimensional geometry of the spine–pelvis via image processing software MIMICS (Materialize Inc., Leuven, Belgium). The three-dimensional geometry was meshed using IAFE-MESH (University of Iowa, Iowa) and HyperMesh (Altair Engineering, Michigan, USA) software. The Abaqus (Dassault Systemes, Simulia Inc., Providence, RI, USA) software was used to assemble the meshed parts and assign material properties to the spine–pelvis model. The vertebrae and pelvis consist of 0.5 mm layer of cortical bone surrounding the cancellous bone. The intervertebral discs comprise of nucleus and annulus with fibers embedded inside it. The ligaments in the model were represented using 2D Truss element formulation in Abaqus. The material properties for all the components were acquired from the literature and are summarized in Table 1.

**Table 1:** Material properties assigned to different components of the finite-element model [2–4]

Structure	Element type	Young’s modulus (MPa)	Poisson’s ratio
Cortical bone	Isotropic, elastic hexahedral elements	12,000	0.3
Cancellous bone	Isotropic, elastic hexahedral elements	100	0.2
Thoracic segment (annulus fibrosus)	Isotropic, elastic hexahedral elements	4.2	0.45
Thoracic segment (nucleus pulposus)	Incompressible fluid, hexahedral elements	9	0.499
Lumbar segment (annulus fibrosus)	Neo Hookian, Hexahedral elements	c10 = 0.348, d1 = 0.3	
Lumbar segment (nucleus pulposus)	Incompressible fluid, hexahedral elements	1	0.4999
Annulus (fibers)	Rebar	357–550	0.3
Anterior longitudinal	Tension-only, truss elements	7.8 (< 12%), 20.0 (> 12%)	0.3
Posterior longitudinal	Tension-only, Truss elements	10.0 (< 11%), 20.0 (> 11%)	0.3
Ligamentum flavum	Tension-only, truss elements	15.0 (< 6.2%), 19.5 (> 6.2%)	0.3
Intertransverse	Tension-only, truss elements	10.0 (< 18%), 58.7 (> 18%)	0.3
Interspinous	Tension-only, truss elements	10.0 (< 14%), 11.6 (> 14%)	0.3
Supraspinous	Tension-only, truss elements	8.0 (< 20%), 15.0 (> 20%)	0.3
Capsular	Tension-only, truss elements	7.5 (< 25%), 32.9 (> 25%)	0.3
Apophyseal joints	Non-linear soft contact, GAPPUNI elements		
Implants			
CoCr (rods)	Isotropic, elastic hexahedral elements	240,000	0.34
Ti (screws, connector, cage)	Isotropic, elastic hexahedral elements	116,000	0.32

### Simulation of different spinopelvic fixations

To simulate the spinopelvic fixations, polyaxial screws were placed from T10–S1/Iliac. The CAD models for screws, w-connector and rods were developed SolidWorks (Dassault Systems, SolidWorks Corporation, Waltham, MA, USA) and meshed in Abaqus. The dimensions of the screws were selected based on vertebral level under the guidance of spine surgeon (T10 and T11: 5.5 × 45 mm; T12–L4: 6.5 × 45 mm; L5 and S1: 7.5 × 45 mm; iliac screw and S2AI screw: 8.5 × 90 mm). Following screws insertion, primary rods were attached to tulip of the polyaxial screws via “TIE” formulation in Abaqus. In some configurations, additional rods (accessory rods) were attached to the primary rod via rod–rod/open–open (i.e., “W”) connectors. For the L5–S1 anterior lumbar interbody fusion (ALIF) cage (titanium; 15 degrees lordosis; anterior height 14 mm; posterior height: 8 mm; width 32 mm; depth 24 mm) was positioned in the center of the interbody space after the entire nucleus of the L5–S1 disc and the anterior 40% of the annulus were removed.

**Fig. 1:**
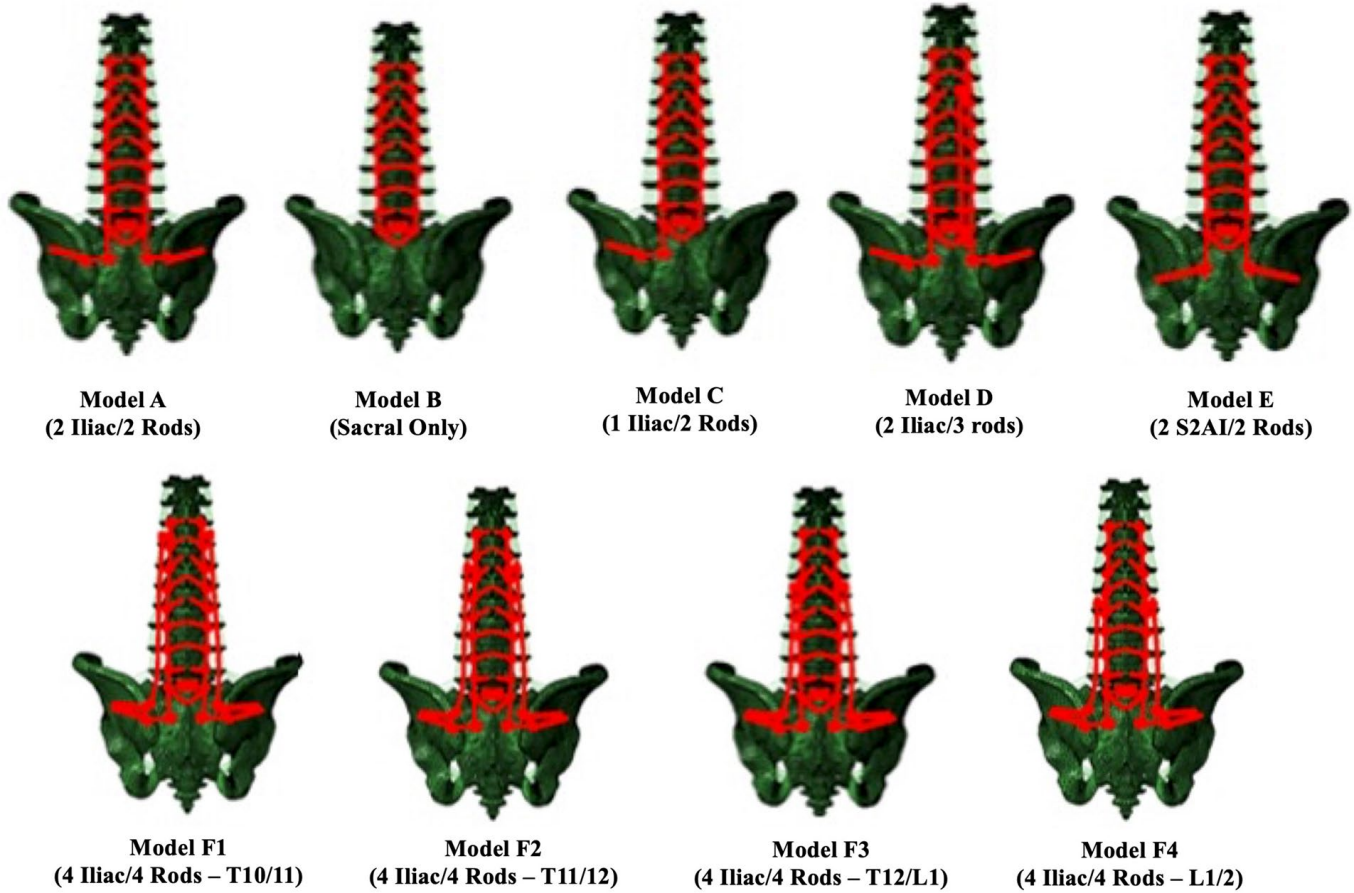
Pictorial representations of each of the 9 posterior instrumentation constructs based on number of rods, type of pelvic fixation, and number of pelvic screw fixation

A total of 9 different T10 to sacrum posterior instrumentation constructs with varying fixation strategies in the pelvis and number of rods were simulated (Fig. 1):

- Model A: one traditional iliac screw bilaterally + 2 main rods (Control: “2 Iliac/2 Rods”).
- Model B: no pelvic fixation + 2 main rods T10 to S1 (“Sacral Only”).
- Model C: unilateral traditional iliac screw + 2 rods (“1 Iliac/2 Rods”).
- Model D: one traditional iliac bolt bilaterally + 2 main rods + one midline accessory rod connected to the midline rod at T11–12 and S1-pelvis (“2 Iliac/3 rods”).
- Model E: one S2AI screw bilaterally + 2 main rods (“2 S2AI/2 Rods”).
- Model F1: two traditional iliac bolts bilaterally + 2 main rods + 2 lateral accessory rods connected to the midline rods at T10–11 and to each proximal iliac bolt (“4 Iliac/4 Rods—T10/11”).

To assess effect of the location of the connection point relative to the proximal junction of the multi-rod constructs, three additional positions of the lateral accessory rod connection point on the midline rods were simulated:

- Model F2: “4 Iliac/4 Rods” with lateral accessory rods connected to midline rods at T11–12 (“4 Iliac/4 Rods—T11/12”).
- Model F3: “4 Iliac/4 Rods” with lateral accessory rods connected to midline rods at T12–L1 (“4 Iliac/4 Rods—T12/L1”).
- Model F4: “4 Iliac/4 Rods” with lateral accessory rods connected to midline rods at L1–2 (“4 Iliac/4 Rods—L1/2”).

### Data analysis

Range of motions (ROM) were calculated by subtracting the rotational angle of upper vertebra from the lower vertebra. The instrumented segments ROM (T10–S1/Iliac) and adjacent segment ROM (T9–T10) were analyzed. The T9–T10 intradiscal pressures and stresses of the T9–10 disc’s annulus in addition to the maximum Von Mises stress on the primary rods, accessory rods, and proximal junction’s vertebral bodies (T9 and T10) were also analyzed. The percentage difference was calculated for all models relative to the Control (Model A: one traditional iliac screw bilaterally + 2 main rods—“2 Iliac/2 Rods”).

## Results

### Range of motions (ROM)

#### T10–S1 ROM (Fig. 2)

**Fig. 2:**
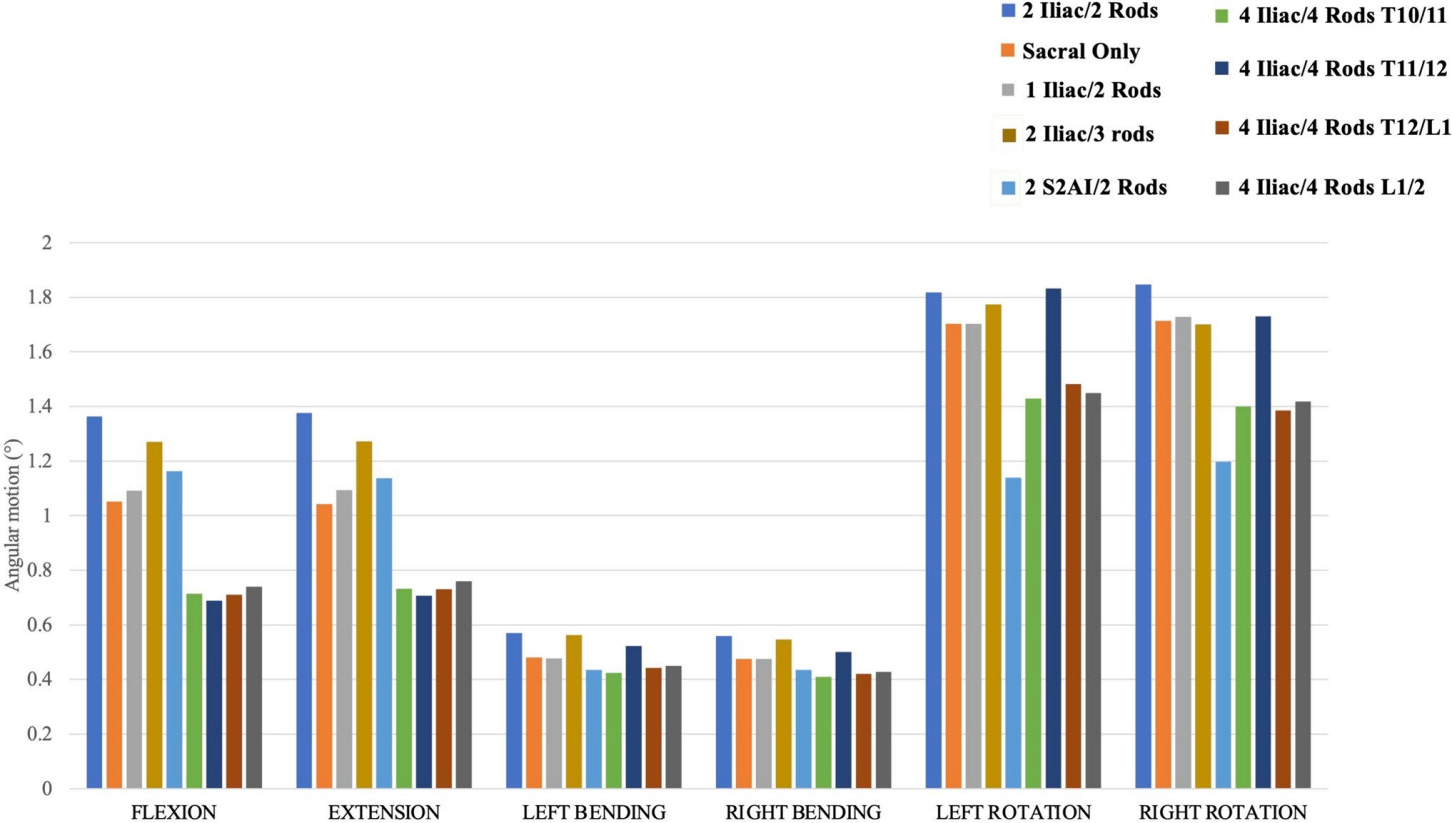
T10–S1 range of motions for each instrumentation construct

T10–S1 range of motion for all instrumentation constructs in any direction of loading was < 2.0 degrees. Left and right bending showed the least range of motion (< 0.6 degrees) for all instrumentation constructs, while the greatest range of motions was found to occur during axial rotation. Under flexion, extension, and side bending (left and right), all models decreased T10–S1 ROM compared to the control. The 4-rod constructs (4 iliac/4 rods) provided the most rigid environment, as they exhibited the greatest reduction in ROMs. In left and right rotation, 2 S2AI/2 Rods exhibited the least ROM followed by the 4-rod constructs (4 iliac/4 rods).

#### T9–T10 ROM (Fig. 3)

**Fig. 3:**
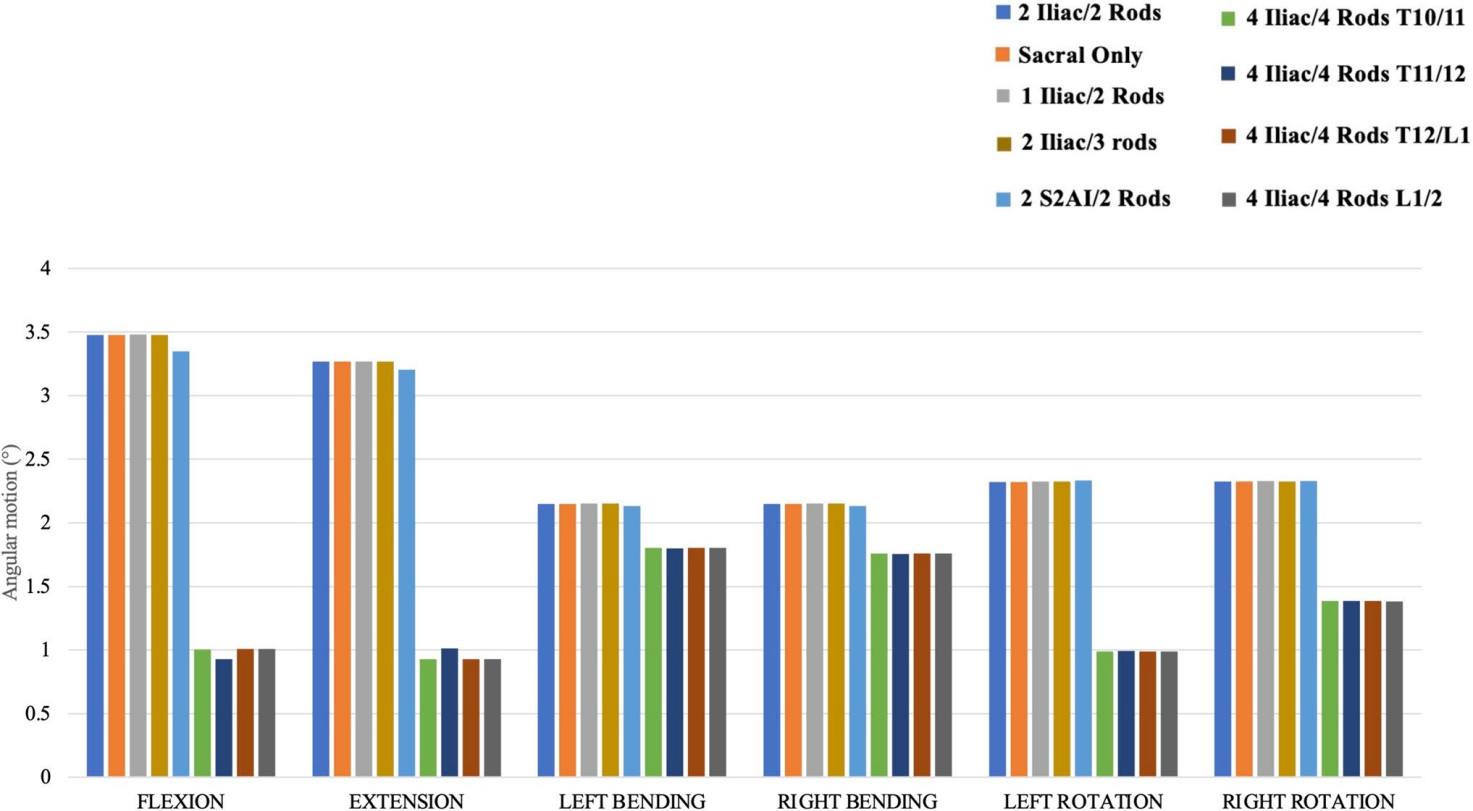
Proximal adjacent segment (T9–10) range of motion for each instrumentation construct

T9–T10 ROM values were similar between the Control (“2 Iliac/2 Rods”) and “Sacral Only”, “1 Iliac/2 Rods”, “2 Iliac/3 rods”, and “2 S2AI/2 Rods”. All four of the 4-rod constructs (4 iliac/4 rods) consistently demonstrated the greatest reduction in ROM across all conditions relative to the control model (flexion: 70.9–73.3% ROM reduction; extension: 69.1–71.6% ROM reduction; left/right bending: 16.1–18.5% ROM reduction; left/right rotation: 40.4–57.4% ROM reduction).

### Von Mises stresses of the proximal junction’s vertebral bodies (T9 and T10)

#### Stresses at T9 (Fig. 4)

**Fig. 4:**
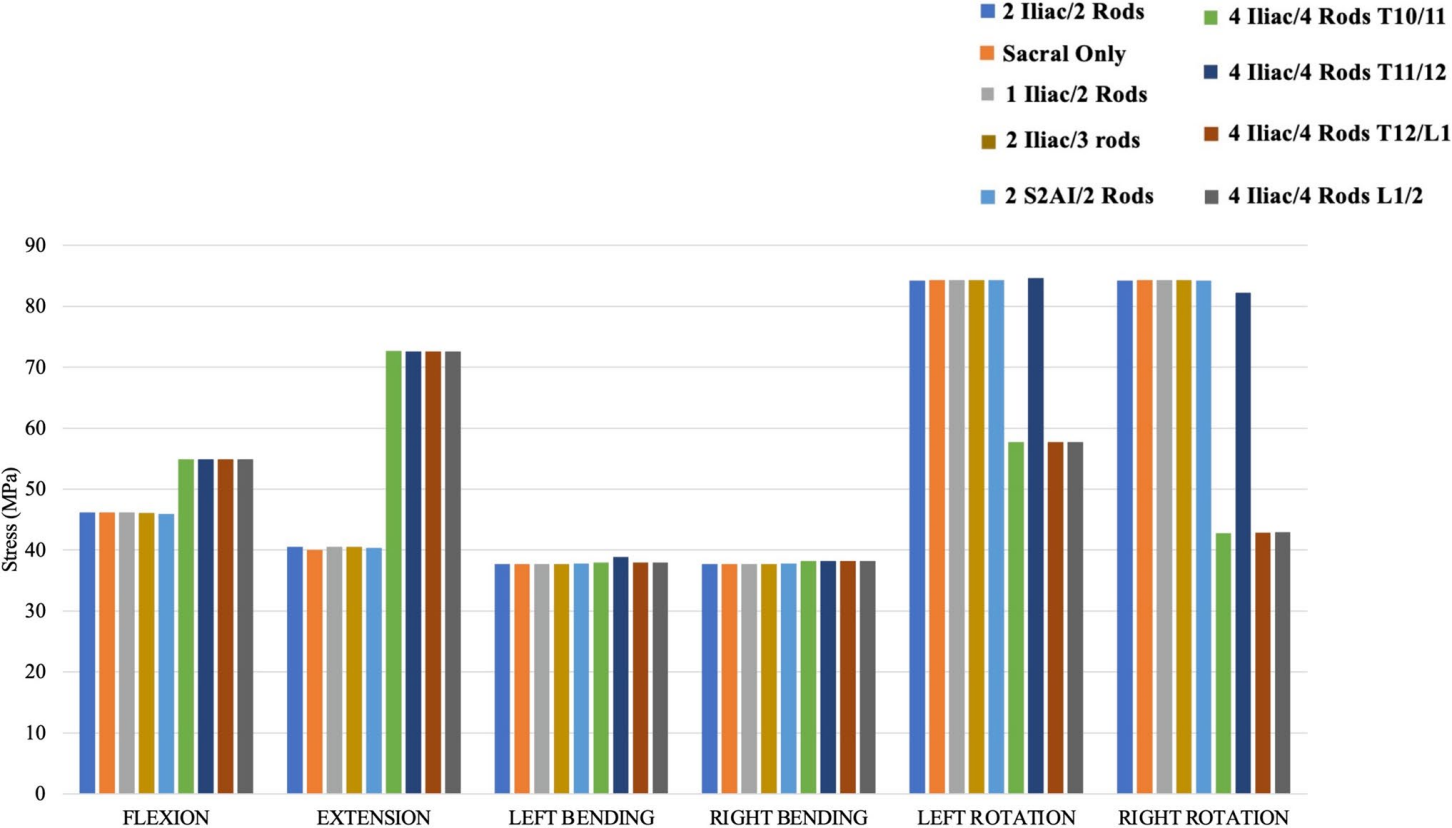
Maximum Von Mises stresses at adjacent segment (T9) vertebral body for each instrumentation construct

Compared to the control model (“2 Iliac/2 Rods”), “Sacral Only”, “1 Iliac/2 Rods”, “2 Iliac/3 rods”, and “2 S2AI/2 Rods” had minimal differences in T9 Von Mises stresses for all six loading conditions. All four variants of the 4-rod constructs exhibited higher maximum Von Mises stresses at the adjacent vertebral body (T9), reaching around 55 MPa for flexion and 72.5 MPa for extension, representing increases of approximately 19% and 79%, respectively, compared to the control. For left and right rotation, all 4-rod construct models, except for 4 iliac/4 rods—T11/12, were noted to decrease rotational stresses at T9 compared to 2-rod and 3-rod constructs. For left and right bending, all models demonstrated consistent maximum stress values between 37.7 MPa and 38.1 MPa, with less than 1.5% difference compared to the control (“2 Iliac/2 Rods”).

#### Stresses at T10 (Fig. 5)

**Fig. 5:**
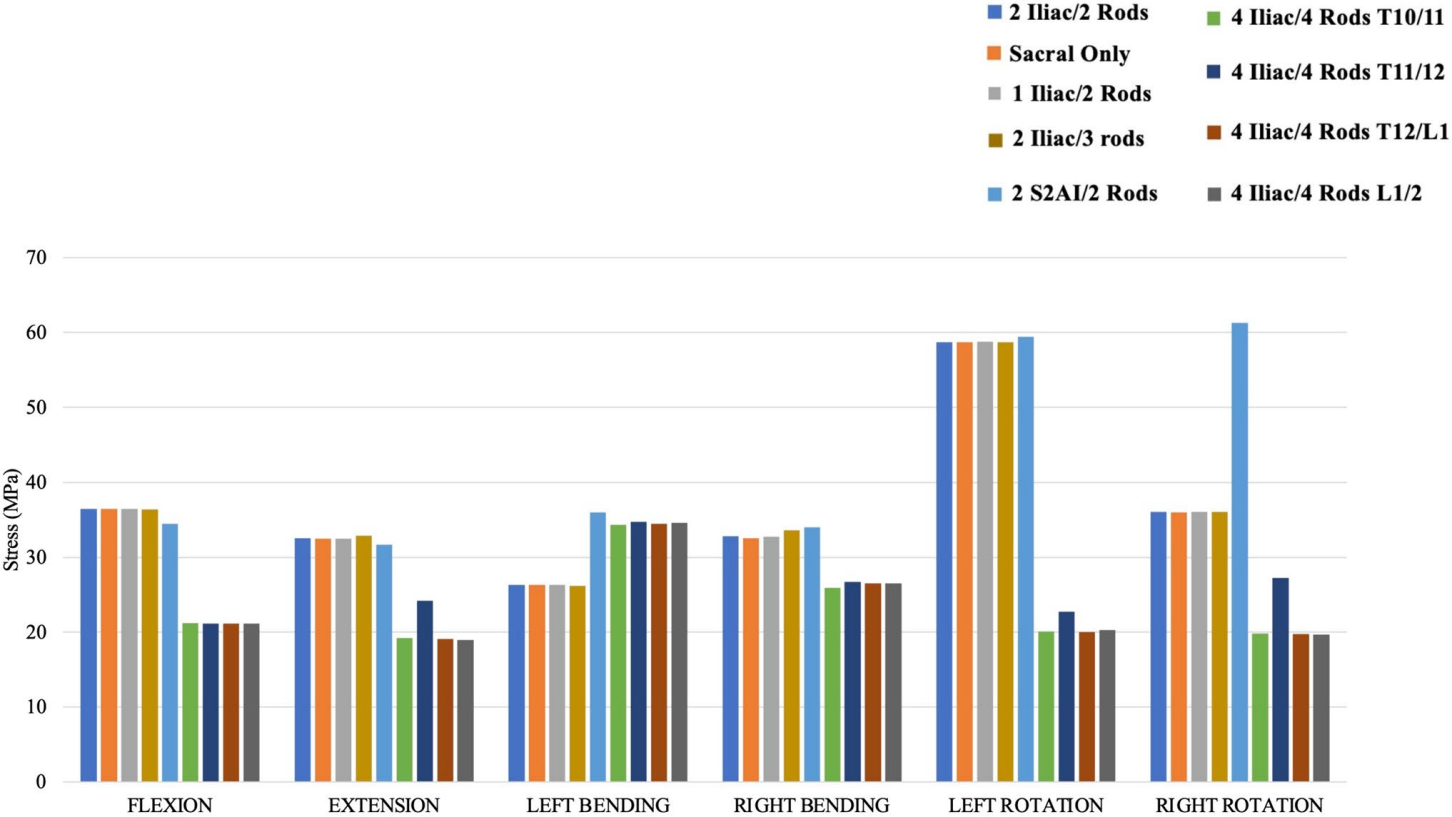
Maximum Von Mises stresses at the upper instrumented vertebra (UIV: T10) for each instrumentation construct

In flexion and extension loading as well as right/left axial rotation loading, “Sacral Only”, “1 Iliac/2 Rods”, “2 Iliac/3 rods”, and “2 S2AI/2 Rods” had similar maximum Von Mises stresses at the T10 vertebral body relative to the Control (“2 Iliac/2 Rods”). All four variants of the 4-rod constructs exhibited the lowest Von Mises stresses at the UIV vertebral body (T10) during flexion loading with values around 21.1 MPa, reflecting the greatest decrease in stress values (41.7–42.0% lower than the control). In addition, all four variants of the 4-rod constructs showed the lowest T10 Von Mises stress values in extension (25.7–41% lower than the control) and right/left axial rotation (24.3–66% lower than the control). Furthermore, stresses at T10 for all 4-rod constructs as well as 2 S2AI/2 Rods were greater in left lateral bending compared to the other 2-rod and 3-rod constructs, while all the 4-rod constructs had lower stresses at T10 in right lateral bending compared to all other 2-rod and 3-rod constructs.

### T9–10 discal parameters

The 4-rod constructs (“4 iliac/rods”) had the lowest intradiscal pressures and lowest annular stresses in flexion and extension relative to the control (Table 2). There were minimal differences between T9–10 discal parameters (intradiscal pressures and annular stresses) for all the other instrumentation configurations relative to the control under all loading conditions (Table 2).

**Table 2:** Maximum Von Mises stresses in nucleus pulposus and annulus fibrosus of adjacent segment (T9–10) disc

	Flexion	Extension	Left bending	Right bending	Left rotation	Right rotation
Von Mises stresses in nucleus pulposus						
Model A (2 iliac/2 rods)	1.338	1.724	1.311	1.324	1.004	1.011
Model B (sacral only)	1.339	1.721	1.311	1.325	1.015	1.018
Model C (1 iliac/2 rods)	1.339	1.72	1.311	1.325	1.017	1.021
Model D (2 iliac/3 rods)	1.339	1.72	1.311	1.325	1.019	1.023
Model E (2 S2AI/2 rods)	1.339	1.72	1.079	1.273	0.8374	0.992
Model F1 (4 iliac/4 rods—T10/11)	1.079	0.727	1.08	1.275	0.8353	0.99
Model F2 (4 iliac/4 rods—T11/T12)	1.079	0.727	1.311	1.325	1.015	1.021
Model F3 (4 iliac/4 rods—T12/L1)	1.079	0.727	1.079	1.272	0.8364	0.9904
Model F4 (4 iliac/4 rods—L1/2)	1.079	0.7272	1.079	1.272	0.8352	0.9892
Von Mises stresses in annulus fibrosus						
Model A (2 iliac/2 rods)	6.02	7.418	4.702	4.657	2.599	2.394
Model B (sacral only)	6.028	7.41	4.746	4.67	2.624	2.392
Model C (1 iliac/2 rods)	6.028	7.41	4.757	4.67	2.624	2.396
Model D (2 iliac/3 rods)	6.028	7.412	4.678	4.755	2.626	2.396
Model E (2 S2AI/2 rods)	6.029	7.414	3.838	5.509	2.624	2.324
Model F1 (4 iliac/4 rods—T10/11)	3.013	2.184	3.837	5.497	2.316	2.613
Model F2 (4 iliac/4 rods—T11/T12)	3.013	2.184	4.669	4.759	2.625	2.395
Model F3 (4 iliac/4 rods—T12/L1)	3.013	2.185	2.185	5.507	2.314	2.606
Model F4 (4 iliac/4 rods—L1/2)	3.012	2.187	3.848	5.521	2.594	2.305

### Rod stresses

#### Primary rod stresses: all models (Fig. 6)

**Fig. 6:**
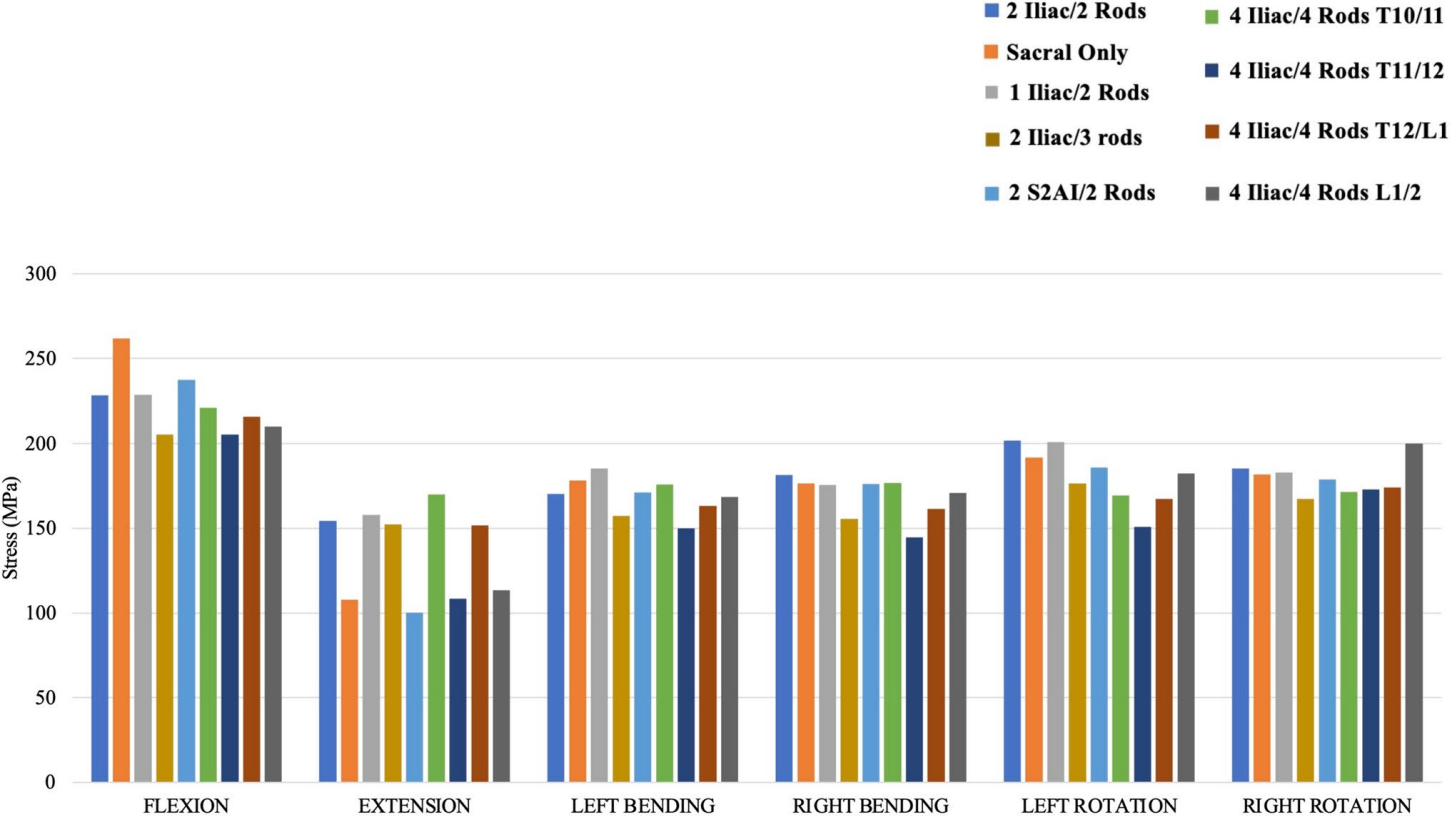
Maximum Von Mises stresses on the primary rods of each instrumentation construct

During flexion, the model without pelvic fixation (“Sacral Only”) exhibited the highest primary rod stresses (262 MPa; 14.8% higher than control), while “4 Iliac/4 Rods—T11/12” and “2 Iliac/3 rods” had the lowest stresses on the primary rods (205.2 MPa; 10.1% less than the control). The other four models had < 5% difference in primary rod Von Mises stresses relative to the control under flexion loading. Under extension, “Sacral Only”, “2 Iliac/3 rods”, “4 Iliac/4 Rods—T11/12”, and “4 Iliac/4 Rods—L1/2” had the lowest Von Mises stresses on the primary rod relative to the control. The other four models had < 5% difference in primary rod Von Mises stresses relative to the control under extension loading. For left and right bending, “4 Iliac/4 Rods—T11/12” and “2 Iliac/3 rods” had the lowest Von Mises stresses on the primary rods relative to the Control and other instrumentation configurations.

#### Accessory rod stresses (Fig. 7)

**Fig. 7:**
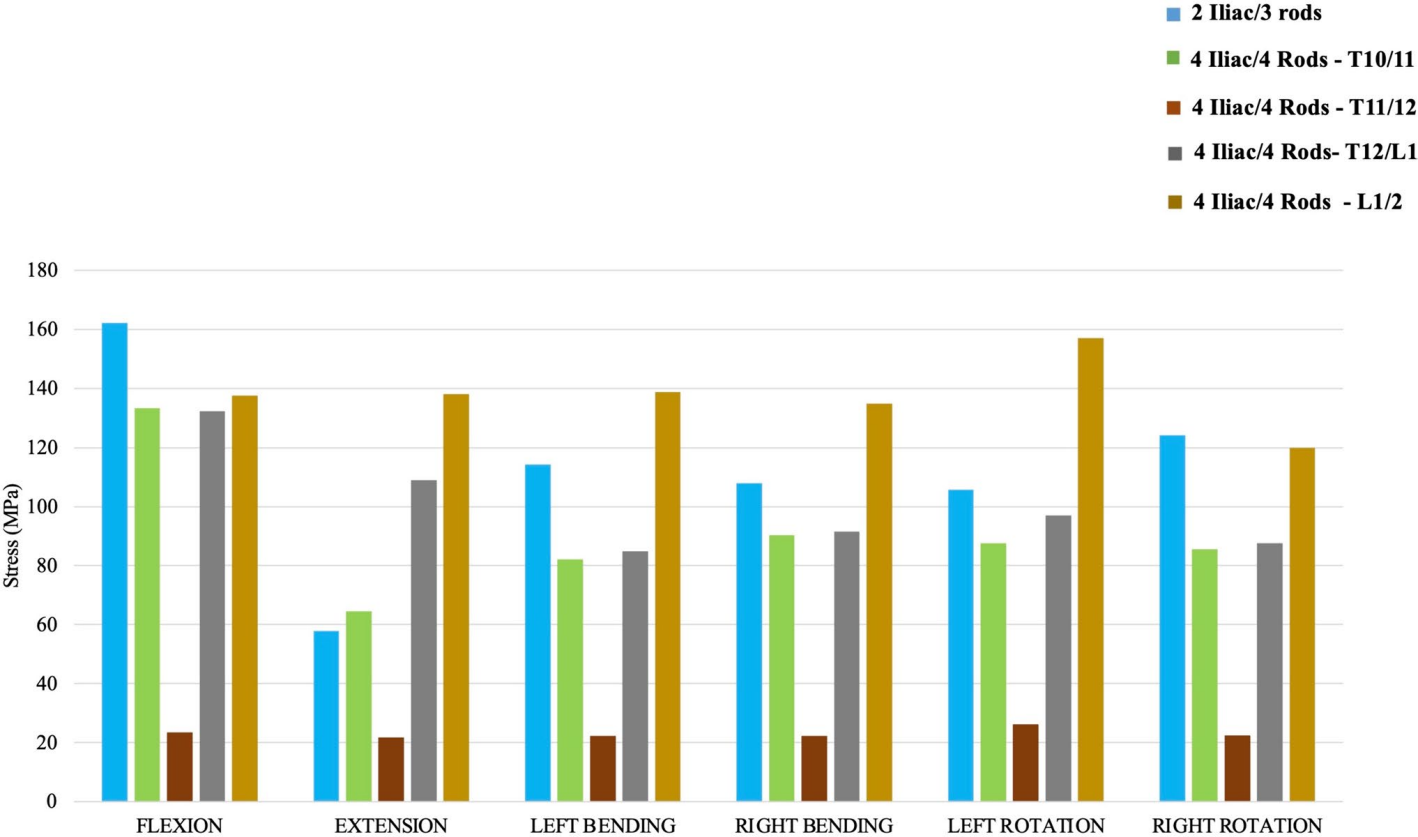
Maximum Von Mises stresses on the accessory rods of each instrumentation construct that was comprised of ≥ *3 rods*

Among the 5 models that included accessory rods, “4 Iliac/4 Rods—T11/12” consistently exhibited the lowest maximum von Mises stresses on the accessory rods across all different loading conditions. In flexion, “2 Iliac/3 rods” had the highest stress value on the accessory rods at 162.1 MPa, while “4 Iliac/4 Rods—T11/12” showed the lowest stress at 23.5 MPa. “4 Iliac/4 Rods—L1/2” had the highest Von Mises stresses on the accessory rods in extension, right/left bending, and right/left axial rotation.

## Discussion

Various techniques for pelvic fixation and multi-rod constructs have proven critical for protecting sacral fixation and reducing complications at the lumbosacral junction and across three-column osteotomies in adult spinal deformity operations. However, the increased rigidity afforded by these constructs raises the question about their differential effects on adjacent segments. While it is theorized that a stiffer construct transmits greater stresses, forces, and range of motion to the proximal junction, the biomechanics of this relationship have not been previously evaluated. As such, in this FE analysis, we assessed the biomechanics of the proximal junction of long thoracolumbar posterior instrumentation constructs commonly used to manage ASD for varying pelvic fixation techniques and number of rods. Our models demonstrated that a multi-rod construct consisting of 4 iliac screws connected to 2 midline rods and 2 lateral accessory rods extending to the thoracolumbar junction had the lowest T10–S1 ROM, lowest adjacent segment disc (T9–T10) ROM, intradiscal pressures, and annular stresses, and lowest UIV (T10) vertebral body stresses. This construct, however, had the highest UIV + 1 (T9) vertebral body stresses compared to the other models. An additional finding was that the location at which the accessory rods terminated relative to the UIV did not have an appreciable effect on the adjacent segment’s biomechanical environment in flexion, extension, and lateral bending.

In long constructs for ASD correction, spinopelvic fixation is often required due to increased force distribution through the lumbosacral junction. There are various techniques to achieve spinopelvic fixation, including the commonly utilized iliac screws and S2AI screws. Burns et al*.* have demonstrated that there is no biomechanical difference in stiffness or load-to-failure between iliac screws and S2AI screws [22]. Both iliac screws and S2AI screws have demonstrated long-term success in achieving fusion in long constructs [23, 24]. In this study, we uniquely demonstrate that different types of pelvic fixation (no pelvic fixation vs. iliac screw unilateral vs. iliac screw bilateral vs. S2 AI bilaterally), while controlling for spinal alignment, do not have different biomechanical effects on the proximal junction of long thoracolumbar posterior instrumentation constructs. While future clinical studies may be beneficial to evaluate the connection between different pelvic fixation strategies and proximal junctional kyphosis/failure, teasing out this relationship may prove challenging given the multitude of patient factors and radiographic parameters involved in proximal junctional pathology in adult spinal deformity. An additional subtle, but interesting finding related to type of pelvic fixation was that bilateral iliac screws combined with 2 or 3 rods were found to have slightly greater T10–S1 ROMs than constructs without pelvic fixation or with only one iliac screw. While the true reasons for these differences are not clear, we speculate that without pelvic fixation, some ROM may be transferred outside of the construct (i.e., the pelvis/SI joints). A more comprehensive understanding of this notion would require a more extensive analysis of segmental ROM within the construct and of the pelvis, which is outside the scope of this study.

In addition to evaluating different pelvic fixation strategies, we aimed to assess the impact of multi-rod constructs on biomechanics of the proximal junction. Multi-rod constructs may be utilized in several clinical scenarios to augment posterior instrumentation [25]. They may be achieved using satellite rods (rods not connected to the main rods), which is a common technique to span a lumbar pedicle subtraction (PSO) or with accessory rods (rods connected to the main rod) that span a 3-column osteotomy site, the lumbosacral junction, and/or thoracolumbar junction and may or may not be attached to pelvic fixation [25]. Accessory rods have gained particular popularity in their use in the kickstand technique for correction of coronal malalignment [26–29]. Irrespective of the strategy, multi-rod constructs have demonstrated efficacy in reducing rates of nonunion and rod breakage across the lumbosacral junction and lumbar PSOs when treating ASD patients undergoing long thoracolumbar instrumented fusion to the pelvis [12, 30].

The utility of multi-rod constructs lies in the locally increased stiffness they create. Despite this regional advantage, it raises the question of whether locally increased stiffness effects a remote site (i.e., the proximal junction). In a large retrospective multicenter study, Gupta et al*.* reported that use of multiple rods in ASD surgery did not result in an increased incidence of PJK [31]. However, as the study evaluated a heterogeneous cohort with respect to type of multi-rod construct (i.e., satellite vs. accessory rods), locations where the additional rods terminated relative to the proximal junction, and level of the upper instrumented vertebrae (i.e., upper thoracic vs. lower thoracic), it is difficult to understand the true connection between multi-rod configurations and the proximal junction of long thoracolumbar instrumentation constructs. In our study, we attempted to clarify this relationship through a variety of multi-rod techniques using only accessory rods that varied based on whether they were connected to pelvic fixation as well as their spinal level relative to the proximal junction. We found that 2-rod constructs had similar adjacent segment biomechanics to a 3-rod construct in which the 3rd rod was an intra-construct accessory rod (i.e., connected to the main rod between the S1 pedicle screw and lateral connector to the pelvis), but different adjacent segment range of motions compared to 4-rod constructs consisting of 4 traditional iliac screws connected to 2 main rods and 2 accessory lateral rods extending to the thoracolumbar junction. We postulate that the 3-rod construct’s effect on the adjacent segment biomechanics is similar to the 2-rod constructs given that the 3rd rod is not connected to an independent pelvic screw, which is in contrast to the 4-rod constructs’ accessory rods that attach to the pelvic screws directly.

We specifically found that the 4-rod constructs demonstrated lower adjacent segment (T9–T10) ROM, lower T9–10 intradiscal pressures and annular stresses, as well as lower UIV (T10) bone stresses. However, they showed the highest UIV + 1 (T9) bone stresses. That the T9–10 ROM and discal parameters were decreased relative to other constructs may suggest the 4-rod constructs provide a more gradual transition of motion between fused and unfused proximal segments (i.e., a “softer landing”) thereby decreasing the risk of PJK/PJF. Alternatively, the decreased adjacent segment motion combined with the finding of increased stresses in the T9 vertebral body may suggest that the 4-rod constructs focus stress on the T9 vertebral body thereby increasing the risk of osseous failure/fractures of the UIV + 1. As these are postulations, further studies are needed to confirm the clinical effects of these biomechanical findings. Other interesting and curious findings were that the location at which the accessory rods ended cranially did not appear to have a measurable effect on the adjacent segment’s biomechanical environment, except for axial rotation in one construct. When the accessory rod terminated at T11–12, it had the lowest Von Mises stresses and resulted in different ROMs in axial rotation and lateral bending through T10–S1 as well as different T9 and T10 vertebral body von Mises stresses in axial rotation compared to the other 4-rod constructs that terminated at T10–11, T12–L1, and L1–2. While our model does not provide reasons for these differences, they may be related to the fact that the connection at T11–12 is immediately across the transition between the thoracic and lumbar spine vertebrae, which differ in shape/anatomy. As the accessory rods stabilize more of the thoracic spine (i.e., T10–11) and/or do not involve the thoracic spine (T12–L1 and L1–2), these biomechanical effects are no longer seen. In addition, it could be secondary to the different locations of the accessory rods in comparison to the primary rods and the relative differences in the number of segments spanned by dual rods.

The results of this study should be considered in the context of its limitations. While the FE framework utilized in this study is well established, its accuracy in capturing the fine nuances of these different instrumentation constructs may be compromised by the fact that the simulations involved no muscle forces and used uncomplicated geometries of the implants and simplified contact. This may be highlighted by the puzzling findings with respect to differences in stresses at the proximal vertebral body for symmetric models, particularly Models A and B, which may be secondary to complex anatomic and/or structural interactions within the model to which we are not privy. Moreover, the residual stresses produced as a result of rod contouring and screw/rod tightening were not considered. Specifically, the interconnections of the screws, rods, rod–rod connectors, and anatomy were all in ideal conditions, which is almost never the case clinically. In addition, primary and accessory rods’ effects on biomechanics may be influenced by other factors, including rod characteristics (i.e., diameter, material, and bend magnitude). While lengths of pedicle screws, S2AI screws, and iliac screws vary in clinical practice based on surgeon preference and patient anatomy, simulation and assessment of different screw lengths for each construct were beyond the scope of this study. In addition, as the non-instrumented spine’s range of motions are not used for comparison, we cannot comment on the significance of the observed changes in range of motion at the proximal adjacent segment relative to normal physiology. Nevertheless, it should be noted that while the model has these limitations, the use of comparative analyses (relative to the Control) make our reported relative differences of greater credence than individual absolute values. While we report relative differences between the different rod configurations, we are unable to comment upon the biomechanical and clinical significances of our observed biomechanical differences and relative long-term clinical performance of the different instrumentation configurations evaluated in this study, particularly because the exact margin of error as well as the margin of important difference are not known. Furthermore, as our study is limited to multi-rod instrumentation constructs consisting of a maximum of 4 rods using traditional iliac screws, the results may not be representative of multi-rod configurations that involve ≥ 5 rods and/or multiple S2AI screws. Although use of more rods is intended to decrease the risk of developing a pseudoarthrosis, the addition of more rods also theoretically may interfere with development and/or maturation of a fusion mass. While an understanding of how much of an adverse effect additional instrumentation has in jeopardizing bony deposition is needed, investigating this is beyond the scope of this study, as it cannot be evaluated by finite-element analysis. Another limitation of this study is that these biomechanical data are derived from a single sagittal plane alignment. While our model consists of a low pelvic incidence lumbar shape with relatively appropriate lumbar lordosis in the upper and lower lumbar spine, our data may be influenced by different shapes of the lumbar spine either emanating from varying pelvic incidences (i.e., low vs. middle vs. high) and/or from sagittal malalignment (i.e., poor lordosis distribution index), particularly given that stress concentrations have been demonstrated to vary according to sagittal plane alignment. Future investigations should ideally aim to address these important questions as well as involve evaluating additional multi-rod constructs using varying combinations and configurations of dual S2AI screws and/or S2AI screws combined with traditional iliac screws. Future studies will also ideally assess SI joint range of motions/stresses as well as variations in biomechanical behavior of these constructs with respect to different physiologic and nonphysiologic lumbar sagittal alignments. Despite these limitations, this is the first study to report the relative effects of various pelvic fixation techniques and number of rods on the biomechanics of the proximal junction of long thoracolumbar posterior instrumentations. As such, we anticipate these data will stimulate future clinical studies and refine our understanding of multi-rod constructs and pelvic fixation in adult spinal deformity operations.

## Conclusions

In this FE analysis, constructs utilizing bilateral dual iliac screws and 4 rods across the lumbosacral junction and extending to the thoracolumbar junction demonstrated the lowest T10–S1 ROM, the lowest adjacent segment disc (T9–T10) ROM, intradiscal pressures, and annular stresses, and the lowest UIV stresses, albeit with the highest UIV + 1 stresses compared to other constructs. Additional studies are needed to confirm whether these biomechanical findings dictate clinical outcomes and effect rates of proximal junctional kyphosis and failure.

## Data Availability

The data that support the findings of this study are available from the corresponding author, AAT, upon reasonable request.
